# Expression of the stem cell markers NANOG and SOX2 in the cervical squamous carcinogenesis

**DOI:** 10.2478/raon-2025-0026

**Published:** 2025-04-24

**Authors:** Miha Koren, Margareta Zlajpah, Mario Poljak, Kristina Fujs Komlos, Margareta Strojan Flezar

**Affiliations:** 1 Institute of Pathology, Faculty of Medicine, University of Ljubljana, Ljubljana, Slovenia; 2 Institute of Microbiology and Immunology, Faculty of Medicine, University of Ljubljana, Ljubljana, Slovenia

**Keywords:** cervical cancer, cervical intraepithelial neoplasia, squamous intraepithelial lesion, cancer stem cell, NANOG, SOX2

## Abstract

**Background:**

The aim of the present study was to assess a diagnostic potential of stem cell markers NANOG and SOX2 for classifying cervical squamous intraepithelial lesions (SILs)/cervical intraepithelial neoplasia (CIN).

**Patients and methods:**

NANOG and SOX2 expression was evaluated immunohistochemically on 40 patients: in 10 cases each of low-grade SIL (LSIL), high-grade SIL/CIN, grade 2 (HSIL/CIN 2), HSIL/CIN, grade 3 (HSIL/CIN 3), cervical squamous cell carcinoma (CSCC) and their adjacent non-dysplastic squamous epithelium. In addition, human papillomavirus (HPV) genotyping and immunohistochemical staining with p16 and Ki-67 were done. NANOG and SOX2 expression was compared between squamous lesions and controls and between squamous lesions by multiplying staining intensity (SI) by the percentage of positive cells (P) and by multiplying SI by the thickness of staining in epithelium (T) to calculate SI x P and SI x T score.

**Results:**

NANOG and SOX2 expression gradually increased from non-dysplastic squamous epithelium via LSIL and HSIL to CSCC. Expression of NANOG and SOX2 was higher in LSIL compared to controls (P < 0.05 for NANOG Si x P and Si x T scores and SOX2 SI x T score) and lower compared to HSIL (P < 0.05 for all SI x P and SI x T scores). HSIL/CIN 3 showed higher SOX2 expression than HSIL/CIN 2 (P < 0.05 for SI x P and SI x T scores).

**Conclusions:**

Contrary to p16, NANOG and SOX2 could be effective for distinguishing LSIL from non-dysplastic changes. NANOG and SOX2 could be surrogate markers for differentiating LSIL from HSIL. Moreover, SOX2 could be helpful for distinguishing HSIL/CIN 2 from HSIL/CIN 3. Further studies with larger numbers of patients and molecular insights are needed.

## Introduction

Cervical cancer is the fourth most common cancer in women worldwide with around 660,000 new cases and 350,000 deaths in 2022.^1^ The highest incidence rates of cervical cancer and over 90% of cervical cancer-related deaths are in low- and middleincome countries.^2^ In high-income countries, cervical cancer incidence and mortality have declined due to human papillomavirus (HPV) vaccination, screening programs and early treatment of precursor lesions.^3,4^

Histologically, precursor lesions of the squamous cervical epithelium are classified as squamous intraepithelial lesions (SILs) or cervical intraepithelial neoplasia (CIN). They are categorized as low-grade SILs (LSILs) encompassing CIN, grade 1 (CIN 1), koilocytic atypia and condyloma acuminatum, and high-grade SILs (HSILs), based on the extension of dysplasia. HSILs may be further subdivided into HSIL/CIN, grade 2 (CIN 2) and HSIL/CIN, grade 3 (CIN 3).^5^ Two-tiered LSIL/HSIL system is preferred because it reflects improved reproducibility and enhanced biological relevance compared to three-tiered CIN 1/2/3 system.^6^ HSIL is caused by transforming infection with high-risk HPV (HR-HPV) that deregulates expression of E6 and E7 viral proteins leading to loss of cell cycle control and uncontrolled cell proliferation.^7,8^

While the majority of LSILs (90%) regress spontaneously, about 10% of LSILs subsequently progress to HSIL. It is estimated that spontaneous regression from HSIL to LSIL occurs in 30% to 50% of cases.^5^ HSIL/CIN 2 lesions show even higher potential for regression.^9^ The risk of progression from untreated HSIL to cancer is 0.5–1% annually, with approximately 30% of HSIL/CIN 3 developing into cervical cancer over a 30-year period.^10^ There are currently no biomarkers that reliably predict the progression of SIL.^11,12^ Imunohistochemical marker p16 is an effective diagnostic tool to differentiate HSIL from mimickers of precancerous lesions. The use of p16 immunohistochemistry is also recommended to clarify the diagnosis of HISL/CIN 2 versus LSIL in morphologically equivocal cases.^6^ Nevertheless, a significant proportion of LSILs show p16 positivity and despite p16 diagnostic value, its staining in LSIL is not predictive for progression of the lesion.^11,12^ Currently, all HSILs are usually surgically excised by large loop excision of the transformation zone (LLETZ), as it is unable to predict which lesion will regress or progress to cervical squamous cell carcinoma (CSCC). Since excision of all HSILs could lead to overtreatment and unnecessary complications related to woman’s reproductive prospect, new markers are needed to predict the natural course of precancerous lesions.^13^

Recent studies on head and neck squamous cell carcinoma (HNSCC) and squamous dysplasia have shown a diagnostic and prognostic potential of a cancer stem cell (CSC) marker NANOG.^14,15^ The relationship between the expression of CSC markers and carcinogenesis of various cancers as well as CSCC has been studied.^16,17^ Increasing evidence has suggested that tumorigenesis depends on CSCs, a small population of cells within a tumour that can self-renew and differentiate into multiple cell types.^16^ CSCs may originate from normal stem cells and express the same markers (CSC markers) as stem cells such as NANOG, OCT3/4, and SOX2.^17^ Several studies have shown that the NANOG protein is highly expressed in cancerous tissues and in early embryonic development, whereas its expression is much lower or absent in normal adult tissues.^18^ Recent data have demonstrated that SOX2 expression is related to several human malignancies and it is nowadays considered as a key driver of squamous carcinogenesis.^19,20^ While several authors reported SOX2 expression in HSIL/CIN 3 and CSCC, only few of them have investigated its expression in LSIL and HSIL/CIN 2.^21–32^ Even less studies have been published regarding NANOG and cervical squamous carcinogenesis.^33–36^

In previous research of CSCs in HNSCC at our institution, more intense NANOG staining was observed during progression of head and neck dysplasia.^37^ The aim of the present study was to assess the ability of CSC markers to discriminate different grades of cervical squamous neoplasia. We evaluated the expression of NANOG and SOX2 using immunohistochemistry in LSIL, HSIL/CIN 2, HSIL/CIN 3, CSCC and their adjacent nondysplastic squamous epithelium. HPV genotyping and immunohistochemical staining with common diagnostic markers p16 and Ki-67 were also performed to confirm the accurate classification of the lesions.

## Patients and methods

### Patients

The study was approved by the National Medical Ethics Committee, Ministry of Health, Republic of Slovenia (consent No. 0120-99/2020/6.) A total of 2,478 cases of SILs and CSCCs, obtained during routine diagnostic and therapeutic procedures from January 2015 to December 2021, were identified from archives of the Institute of Pathology, Medical Faculty, University of Ljubljana, Slovenia. After detailed review, 10 samples of each LSIL, HSIL/CIN 2, HSIL/CIN 3 and CSCCs from total of 40 patients with unequivocal diagnoses and sufficient tissue for further analysis were selected. Their adjacent non-dysplastic squamous epithelium was used as controls for comparison with cervical squamous neoplasia. None of the patients involved received chemotherapy, radiotherapy or immunotherapy before the specimen collection.

### HPV genotyping

All lesions were selected for HPV genotype analysis. Tissue cores from representative areas of formalin-fixed, paraffin-embedded (FFPE) tissue blocks were punched using a 600 μm needle. Total DNA from at least three punches per FFPE sample was extracted by an overnight digestion with protease at 55°C, followed by DNA purification using a MagMax FFPE DNA/RNA Ultra Kit (Applied Biosystems; Thermo Fisher Scientific, Waltham, MA, USA) according to the manufacturer’s instructions with one modification: protease digestion was performed overnight mixing at 300 rpm for 15 seconds every 4 minutes instead of 1 hour. DNA concentrations were estimated by spectrophotometric analysis at 260 nm using the NanoDrop™ 1000 spectrophotometer (Thermo Fisher Scientific, Wilmington, DE, USA). Apart from the deparaffinization solution (xylene; Sigma-Aldrich; Merck KGaA, Darmstadt, Germany) and the ethanol (Merck KGaA, Darmstadt, Germany), all the reagents were from Applied Biosystems (Thermo Fisher Scientific, Waltham, MA, USA).

To determine the presence of HPV, 100 ng of DNA was tested with commercial HPV genotyping test Anyplex™ IIHPV28 Detection (Seegene, Seoul, South Korea), which is capable of simultaneous detection and differentiation of 28 HPV genotypes (HPV-6, 11, 16, 18, 26, 31, 33, 35, 39, 40, 42, 43, 44, 45, 51, 52, 53, 54, 56, 58, 59, 61, 66, 68, 69, 70, 73, and 82).^38^ Each multiplex PGR reaction was performed according to the manufacturer’s instructions.^39^

### Immunohistochemistry and evaluation

Immunohistochemical analysis of pl6, Ki-67, NANOG and SOX2 expression was performed on 3-4 μm thick FFPE tissue sections. After deparaffinization, hydration and automated antigen retrieval (BenchMark ULTRA, Ventana, F. Hoffmann-La Roche (Roche Holding AG), Basel, Switzerland), slides were incubated with commercially available anti-p16 (Ventana (F. Hoffmann-La Roche (Roche Holding AG), Basel, Switzerland), cat no. 805-4713, ready to use) anti-ki67 (Agilent Dako, Santa Clara, California, USA, cat no. M7240, dilution 1:50), anti-NANOG (Cell Signaling Technology, Danvers, Massachusetts, USA, cat no. 4903T, dilution 1 : 100) and anti-SOX2 (Ventana (F. Hoffmann-La Roche (Roche Holding AG), Basel, Switzerland), cat no. 760-4621, ready to use) antibodies. Reactions were visualized by incubation with peroxidase and 3,3′-diaminobenzidine (p16, NANOG and SOX2: OptiVIEW DAB Detection Kit, Roche, Basel, Switzerland; Ki-67: ultraVIEW, DAB Detection Kit, Roche, Basel, Switzerland) and then counterstained with hematoxylin. Negative controls omitting the primary antibody binding were included in every run of samples. Positive controls included HPV-associated oropharyngeal SCC for p16, tonsillar lymphatic tissue for Ki-67, testicular seminoma, oral SCC and HSIL/CIN 3 for NANOG and basal cells of non-dysplastic squamous cervical epithelium as an internal positive control for SOX2.

All slides were evaluated independently by two pathologists (M.S.F. and M.K.) and consensus agreements were reached in discordant cases. Expression of p16 was scored as positive (continuous strong nuclear or nuclear plus cytoplasmic staining of the basal cell layer with extension upward involving at least one third of the epithelial thickness) or negative (any other staining) in dysplastic and non-dysplastic squamous epithelium, as previously described, and as positive (any positive staining) or negative for CSCC.^6^ Expression of Ki-67 in the nuclei was assessed by thickness of staining in squamous epithelium for SIL and controls (0: parabasal cells or no staining, 1: basal one third, 2: basal two third, 3: full thickness) and for CSCC semiquantitatively according to the percentage of positive cells (0: 0%, 1: 1% to 29%, 2: 30% to 59%, 3: 60% to 100%).^29,40^ In all controls, SIL and CSCC, the expression of NANOG and SOX2 was assessed by the percentage of positive cells, staining intensity and staining pattern (nuclear, cytoplasmic, nuclear or cytoplasmic); SOX2 staining intensity and percentage of positive cells were evaluated above the basal layer. Staining intensity of NANOG (0: no staining, 1: weak, 2: moderate, 3: strong - as in testicular seminoma on slide control) and SOX2 (0: no staining, 1: weak, 2: moderate - as basal cells of adjacent non-dysplastic squamous epithelium, 3: strong) was scored in a maximally stained area. Based on previously published studies, the percentage of positive cells was evaluated semiquantitatively and divided into five categories for both NANOG (0: < 5%, 1: 5-25%, 2: 25-50%, 3: 50-75%, 4 > 75%) and SOX2 (0: 0-10%, 1: 10 -25%, 2: 25-50%, 3: 50-75%, 4 > 75%).^21,24,34,37^ In addition, the expression of NANOG (0: no staining, 1: basal one third, 2: basal two third, 3: full thickness) and SOX2 (0: basal cells or no staining, 1: basal one third, 2: basal two third, 3: full thickness) in SIL and controls was assessed based on the thickness of staining in squamous epithelium.^29^ For both NANOG and SOX2, staining intensity and percentage of positive cells (SIxP score) as well as staining intensity and thickness of staining (SIxT score) were multiplied to calculate SIxP score and SIxT score.^21,24,34^

### Statistical analysis

Statistical analysis was performed using IBM SPSS Statistics 24.0 software (IBM Corp.). The Mann-Whitney U test was used and differences were considered as statistically significant at cut-off p ≤ 0.05 (2-tailed).

## Results

### Patients

The mean age of patients in the LSIL group was 47 years, 37 years in the HSIL/CIN 2 group, 41.1 years in the HSIL/CIN 3 group and 47.6 years in the CSCC group. One out of 10 LSILs was obtained by hysterectomy, all other SILs were excised by cone biopsy (9 cases) or LLETZ (20 cases). In the CSCC group, 1 of 10 samples was a hysterectomy, 2 of 10 were cervical biopsies, 2 of 10 were LLETZs and 5 of 10 were cone biopsies. In 37 of 40 cases, adjacent non-dysplastic squamous epithelium under hormonal stimulation (normal reproductive age ectocervical epithelium) was found and used as control. In a case of HSIL/CIN 2 and in a case of HSIL/CIN 3, adjacent squamous metaplastic epithelium was used as their controls and in a case of CSCC adjacent atrophic squamous epithelium was used as a control.

### HPV genotyping

The presence of HPV DNA was tested and confirmed in all 40 patients included in the study. Distribution of HPV genotypes according to histological diagnosis is shown in Table 1. Single HPV genotype infection was found in 1 of 10 LSILs, 7 of 10 HSIL/CIN 2 cases, 8 of 10 HSIL/CIN 3 cases and 6 of 10 CSCCs. Coinfection with two or more HPV genotypes was found in 9 of 10 LSILs, 3 of 10 HSIL/CIN 2 cases, 2 of 10 HSIL/CIN 3 cases and 4 of 10 CSCCs. In 2 LSILs and 1 HSIL/CIN 2 cases, only possibly carcinogenic HPV genotypes 66 and 70 and no HR-HPV genotypes were present. The remaining 37 patients were infected with HR-HPV genotypes 16, 18, 31, 39, 51, 52, 56, 58 and 59.^7,41^ HPV-16 was the most common genotype, followed by HPV-31, HPV-51 and HPV-66.

**TABLE 1.** Results of human papillomavirus (HPV) genotyping in squamous lesions

HPV type	HPV 16	HPV 18	HPV 31	HPV 39	HPV 51	HPV 52	HPV 53	HPV 56	HPV 58	HPV 59	HPV 68	HPV 66	HPV 70	HPV 42
LSIL, n	4	0	0	3	2	0	3	1	0	1	2	5	2	2
HSIL/CIN 2, n	4	1	1	0	1	2	0	1	2	0	0	1	0	0
HSIL/CIN 3, n	8	0	3	0	0	0	1	0	0	1	0	0	0	0
CSCC, n	6	2	2	0	3	0	0	0	0	0	0	0	1	0

1 CSCC = cervical squamous cell carcinoma; HPV = human papillomavirus; HSIL/CIN 2 = high-grade squamous intraepithelial lesion/cervical intraepithelial neoplasia grade 2; HSIL/CIN 3 = high-grade squamous intraepithelial lesion/cervical intraepithelial neoplasia grade 3; LSIL = low-grade squamous intraepithelial lesion; n = number of cases

### Immunohistochemistry

**Figure 1.**
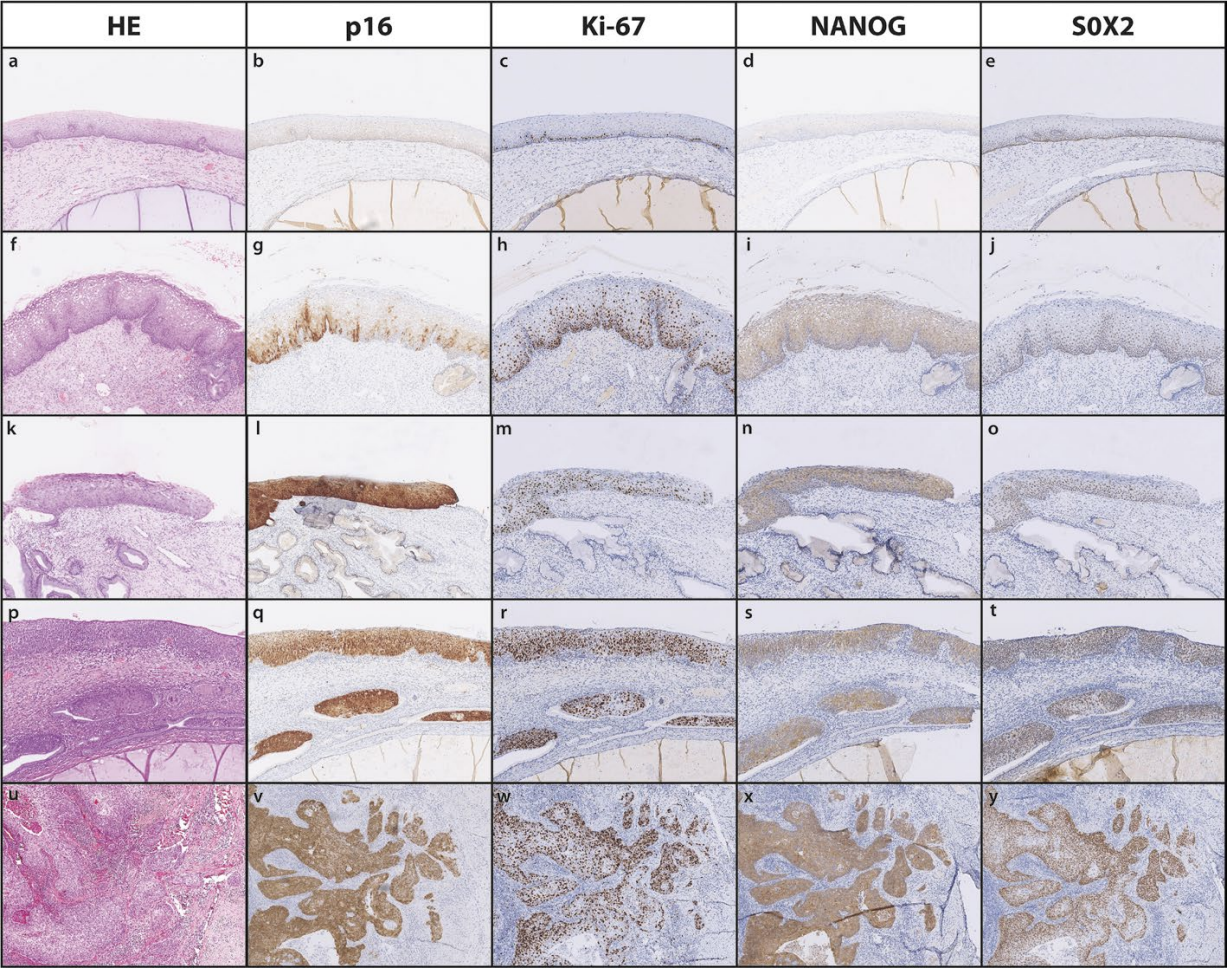
Representative images of p16 (b, g, l, q, v), Ki-67 (c, h, m r, w), NANOG (d, i, n, s, x) and SOX2 (e, j, o, t, y) immunohistochemical staining in a control (a-e), low-grade squamous intraepithelial lesion (LSIL) (f-j), high-grade squamous intraepithelial lesion/cervical intraepithelial neoplasia grade 2 (HSIL/CIN 2) (k-o), high-grade squamous intraepithelial lesion/cervical intraepithelial neoplasia grade 3 (HSIL/CIN 3) (p-t) and cervical squamous cell carcinoma (CSCC) (u-y). HE = haematoxylin and eosin stain

Immunohistochemical analysis of p16, Ki-67, NANOG and SOX2 was performed for all selected samples. Representative images for each group are presented in Figure 1. All 40 included control tissues were immunohistochemically negative for p16 and expression of Ki-67 was limited on the parabasal layer. Two of 10 LSILs were positive for p16 and 2 of 10 LSILs were negative for p16. Six of 10 LSILs were negative for p16 in the major part of the lesion and positive for p16 in the minor part of the lesion. All 20 cases of HSIL and all 10 cases of CSCC were positive for p16 and showed full thickness and/or overall expression of Ki-67. In LSIL, Ki-67 expression was in full thickness of epithelium in 8 out of 10 cases and predominantly in the basal two thirds of epithelium in 2 out of 10 cases.

Immunohistochemical staining for NANOG was present in the cytoplasm of squamous cells; no nuclear staining was observed. In general, controls showed lower staining intensity, percentage and thickness of staining of NANOG than their adjacent SIL and CSCC (Table 2). No reaction was observed in 3 of 40 (7.5 %) controls; one of them was an atrophic squamous epithelium and two were normal reproductive age ectocervical epithelium. In other controls, staining intensity was mostly weak (in 80 %) and extending up to basal two thirds of epithelium (in 87.5%), with less than 50% of cells being positive in 31 of 40 controls. In a majority of LSILs, staining intensity was moderate (in 70%) and positive in more than 75% of cells (in 90%), with full thickness expression observed in all cases. Moderate or strong staining intensity was found in 9 out of 10 cases of HSIL/CIN 2 and in all 10 cases of HSIL/CIN 3. More than 75% of positive cells and full thickness staining was observed in the majority of HSIL/CIN 2 (in 90%) and in all HSIL/CIN 3. The reaction was mostly strong (in 90%) and in more than 75% of cells in all cases of CSCC.

**TABLE 2.** Evaluation of NANOG staining in controls and squamous lesions

	NANOG SI, n (%)				NANOG P, n (%)					NANOG T, n (%)			
Score	0	1	2	3	0	1	2	3	4	0	1	2	3
Controls (n = 40)	3 (7.5)	32 (80)	5 (12.5)	0 (0)	3 (7.5)	6 (15)	22 (55)	8 (20)	1 (2.5)	3 (7.5)	4 (10)	28 (70)	5 (12.5)
LSIL (n = 10)	0 (0)	2 (20)	7 (70)	1 (10)	0 (0)	0 (0)	0 (0)	1 (10)	9 (90)	0 (0)	0 (0)	0 (0)	10 (100)
HSIL/CIN 2 (n = 10)	1 (10)	0 (0)	4 (40)	5 (50)	1 (10)	0 (0)	0 (0)	0 (0)	9 (90)	1 (0)	0 (0)	0 (0)	9 (90)
HSIL/CIN 3 (n = 10)	0 (0)	0 (0)	1 (10)	9 (90)	0 (0)	0 (0)	0 (0)	0 (0)	10 (10)	0 (0)	0 (0)	0 (0)	10 (100)
CSCC (n = 10)	0 (0)	0 (0)	1 (10)	9 (90)	0 (0)	0 (0)	0 (0)	0 (0)	10 (100)	Not applicable^1^			

1 CSCC = cervical squamous cell carcinoma; HSIL/CIN 2 = high-grade squamous intraepithelial lesion/cervical intraepithelial neoplasia grade 2; HSIL/CIN 3 = high-grade squamous intraepithelial lesion/cervical intraepithelial neoplasia grade 3; LSIL = low-grade squamous intraepithelial lesion; n = number of cases; P = percentage of positive cells; SI = staining intensity for NANOG; T = thickness of staining; % = percentage of all cases in each category

1 T was not evaluated in CSCC.

Immunohistochemical staining for SOX2 was observed only in the nuclei of squamous cells. SIL and CSCC showed higher staining intensity, percentage and thickness of SOX2 staining than their adjacent normal squamous epithelium (Table 3). Moderate intensity of staining in the basal cells was present in 37 of 40 controls (92.5%). Weak SOX2 reaction was observed in basal and upper cells of an atrophic squamous epithelium. Complete absence of SOX2 staining was in the two cases of control squamous metaplastic epithelium. Staining intensity was mostly weak (in 82.5%), with less than 50% positive cells (in 72.5%) and extending up to basal two thirds of epithelium (in 85%) in other controls. Majority of LSILs showed moderate staining intensity (in 60%) in more than 50% of cells (in 80%), extending at least up to basal two thirds of epithelium in all cases. In HSIL/CIN 2, moderate staining intensity (in 70%), involving full thickness of epithelium (in 90%), was mostly observed, with more than half of positive cells in all cases. Majority of HSIL/CIN 3 (in 80%) and CSCC (in 70%) strongly expressed SOX2 in more than 75% of cells (in 80%). In all HSIL/CIN 3, full thickness of epithelium was involved.

**TABLE 3.** Evaluation of SOX2 staining in controls and squamous lesions

	SOX2 SI, n (%)				SOX2 P, n (%)					SOX2 T, n (%)			
Score	0	1	2	3	0	1	2	3	4	0	1	2	3
Controls (n = 40)	2 (5)	33 (82.5)	5 (12.5)	0 (0)	2 (5)	1 (2.5)	26 (65)	10 (25)	0 (0)	2 (5)	1 (2.5)	31 (77.5)	6 (15)
LSIL (n = 10)	0 (0)	4 (40)	6 (60)	0 (0)	0 (0)	0 (0)	2 (20)	4 (40)	4 (40)	0 (0)	0 (0)	4 (40)	6 (60)
HSIL/CIN 2 (n = 10)	0 (0)	1 (10)	7 (70)	2 (20)	0 (0)	0 (0)	0 (0)	4 (40)	6 (60)	0 (0)	0 (0)	1 (10)	9 (90)
HSIL/CIN 3 (n = 10)	0 (0)	1 (10)	1 (10)	8 (80)	1 (10)	0 (0)	0 (0)	1 (10)	8 (80)	0 (0)	0 (0)	0 (0)	10 (100)
CSCC (n = 10)	0 (0)	0 (0)	3 (30)	7 (70)	0 (0)	0 (0)	0 (0)	2 (20)	8 (80)	Not applicable^1^			

1 CSCC = cervical squamous cell carcinoma; HSIL/CIN 2 = high-grade squamous intraepithelial lesion/cervical intraepithelial neoplasia grade 2; HSIL/CIN 3 = high-grade squamous intraepithelial lesion/cervical intraepithelial neoplasia grade 3; LSIL = low-grade squamous intraepithelial lesion; n = number of cases; P = percentage of positive cells; SI = staining intensity for NANOG; T = thickness of staining; % = percentage of all cases in each category

1 T was not evaluated in CSCC.

### Comparison between non-dysplastic squamous epithelia and squamous lesions

Statistical analysis of SIxP score and SIxT score for NANOG and SOX2 for controls, LSIL, HSIL/CIN 2, HSIL/CIN 3 and CSCC was performed and results are summarized in Table 4. Both NANOG scores were statistically significantly higher in LSIL, HSIL/CIN 2, HSIL/CIN 3 and CSCC compared to their adjacent non-dysplastic squamous epithelium. LSIL, HSIL/CIN 2, HSIL/CIN 3 and CSCC also showed higher scores for SOX2 than their adjacent control epithelium. The difference was statistically significant in all cases except when LSIL SIxP score was compared to their controls. However, even in this case, it was very close to the cut-off value of p ≤ 0.05 (p = 0.054).

**TABLE 4.** Comparison of NANOG and SOX2 staining between squamous lesions and their adjacent control non-dysplastic squamous epithelium

NANOG SIxP score, a ± SD		p-value^1^	SOX2 SIxP score, a ± SD		p-value^1^
C (LSIL)	LSIL		C (LSIL)	LSIL	
2.30 ± 0.95	7.50 ± 2.46	0.000	3.00 ± 1.41	5.40 ± 2.63	0.054
C (HSIL/CIN 2)	HSIL/CIN 2		C (HSIL/CIN 2)	HSIL/CIN 2	
3.30 ± 2.06	9.20 ± 3.79	0.002	2.20 ± 1.48	7.70 ± 2.75	0.000
C (HSIL/CIN 3)	HSIL/CIN 3		C (HSIL/CIN 3)	HSIL/CIN 3	
2.00 ± 0.94	10.40 ± 2.07	0.000	2.30 ± 1.16	10.10 ± 3.84	0.002
C (CSCC)	CSCC		C (CSCC)	CSCC	
1.44 ± 1,01	11.56 ± 1.33	0.000	2.44 ± 0,53	10.22 ± 2.73	0.000
C (HSIL)	HSIL		C (HSIL)	HSIL	
2.65 ± 1.65	9.80 ± 2.96	0.000	2.25 ± 1.26	8.90 ± 3.39	0.000

NANOG SIxT score^2^ a ± SD		p-value^1^	SOX2 SIxT score^2^, a ± SD		p-value^1^
C (LSIL)	LSIL		C (LSIL)	LSIL	
1.90 ± 0.32	5.70 ± 1.70	0.000	2.50 ± 0.85	4.30 ± 1.89	0.0027
C (HSIL/CIN 2)	HSIL/CIN 2		C (HSIL/CIN 2)	HSIL/CIN 2	
3.00 ± 1.76	6.90 ± 2.85	0.005	1.90 ± 0.99	6.10 ± 1.85	0.000
C (HSIL/CIN 3)	HSIL/CIN 3		C (HSIL/CIN 3)	HSIL/CIN 3	
2.20 ± 1.48	7.80 ± 1.55	0.000	2.40 ± 1.17	8.10 ± 2.02	0.000
C (HSIL)	HSIL		C (HSIL)	HSIL	
2.60 ± 1.59	7.35 ± 2.22	0.000	2.15 ± 1.06	7.10 ± 2.10	0.000

1 a = average; C (HSIL) = control non-dysplastic squamous epithelium adjacent to high-grade squamous intraepithelial lesion; C (LSIL) = control non-dysplastic squamous epithelium adjacent to low-grade squamous intraepithelial lesion; C (HSIL/CIN 2) = control non-dysplastic squamous epithelium adjacent to high-grade squamous intraepithelial lesion/cervical intraepithelial neoplasia grade 2; C (HSIL/CIN 3) = control non-dysplastic squamous epithelium adjacent to high-grade squamous intraepithelial lesion/cervical intraepithelial neoplasia grade 3; CSCC = cervical squamous cell carcinoma; C (CSCC) = control non-dysplastic squamous epithelium adjacent to cervical squamous cell carcinoma; HSIL = high-grade squamous intraepithelial lesion; HSIL/CIN 2 = high-grade squamous intraepithelial lesion/cervical intraepithelial neoplasia grade 2; HSIL/CIN 3 = high-grade squamous intraepithelial lesion/cervical intraepithelial neoplasia grade 3; LSIL = low-grade squamous intraepithelial lesion; SD = standard deviation; SIxP score = staining intensity (SI) multiplied by percentage of positive cells (P); SIxT score = staining intensity (SI) multiplied by thickness of staining (T)

1 Comparisons between groups were tested by the Mann-Whitney test.

2 SIxT score was not calculated for CSCC because T was not evaluated in CSCC.

### Comparison between squamous lesions

Comparison of SIxP score and SIxT score for NANOG and SOX2 between lesions was statistically analysed and results are presented in Table 5. NANOG scores were higher in CSCC compared to HSIL/CIN 3 and HSIL, in HSIL/CIN 3 compared to HSIL/CIN 2, in HSIL/CIN 2 compared to LSIL and in HSIL compared to LSIL. However, the difference was statistically significant only between HSIL and LSIL. SOX2 scores were also higher in CSCC compared to HSIL/CIN 3 and HSIL, in HSIL/CIN 3 compared to HSIL/CIN 2, in HSIL/CIN 2 compared to LSIL and in HSIL compared to LSIL. Statistically significant difference was between HSIL and LSIL and, interestingly, also between HSIL/CIN 3 and HSIL/CIN 2.

**TABLE 5.** Comparison of NANOG and SOX2 staining between squamous lesions

NANOG SIxP score, a ± SD		p-value^1^	SOX2 SIxP score, a ± SD		p-value^1^
LSIL	HSIL/CIN 2		LSIL	HSIL/CIN 2	
7.50 ± 2.46	9.20 ± 3.79	0.101	5.40 ± 2.63	7.70 ± 2.75	0.114
HSIL/CIN 2	HSIL/CIN 3		HSIL/CIN 2	HSIL/CIN 3	
9.20 ± 3.79	10.40 ± 2.07	0.547	7.70 ± 2.75	10.10 ± 3.84	0.031
HSIL/CIN 3	CSCC		HSIL/CIN 3	CSCC	
10.40 ± 2.07	11.56 ± 1.33	0.165	10.10 ± 3.84	10.22 ± 2.73	0.805
LSIL	HSIL		LSIL	HSIL	
7.50 ± 2.46	9.80 ± 2.96	0.018	5.40 ± 2.63	8.90 ± 3.39	0.009
HSIL	CSCC		HSIL	CSCC	
9.80 ± 2.96	11.56 ± 1.33	0.078	8.90 ± 3.39	10.22 ± 2.73	0.370

NANOG SIxT score^2^ a ± SD		p-value^1^	SOX2 SIxT score^2^, a ± SD		p-value^1^
LSIL	HSIL/CIN 2		LSIL	HSIL/CIN 2	
5.70 ± 1.70	6.90 ± 2.85	0.101	4.30 ± 1.89	6.10 ± 1.85	0.056
HSIL/CIN 2	HSIL/CIN 3		HSIL/CIN 2	HSIL/CIN 3	
6.90 ± 2.85	7.80 ± 1.55	0.547	6.10 ± 1.85	8.10 ± 2.02	0.023
LSIL	HSIL		LSIL	HSIL	
5.70 ± 1.70	7.35 ± 2.22	0.018	4.30 ± 1.89	7.10 ± 2.10	0.003

1 a = average; CSCC = cervical squamous cell carcinoma; HSIL = high-grade squamous intraepithelial lesion; HSIL/CIN 2 = high-grade squamous intraepithelial lesion/cervical intraepithelial neoplasia grade 2; HSIL/CIN 3 = high-grade squamous intraepithelial lesion/cervical intraepithelial neoplasia grade 3; LSIL = low-grade squamous intraepithelial lesion; SD = standard deviation; SIxP score = staining intensity (SI) multiplied by percentage of positive cells (P); SIxT score = staining intensity (SI) multiplied by thickness of staining (T)

1 Comparisons between groups were tested by the Mann-Whitney test.

2 SIxT score was not calculated for CSCC because T was not evaluated in CSCC.

## Discussion

According to the current knowledge, CSCs are the key factor in tumour initiation and development, metastasis, and recurrence.^16^ CSCs and normal stem cells share similar signalling pathways and transcription factors, including NANOG and SOX2.^17^ These proteins have been found overexpressed in cells of variety of precancerous and cancerous lesions, exhibiting an important function during carcinogenesis. By expressing NANOG and SOX2 among the others, these cells show CSC-like properties.^42,43^ In the present study, we demonstrated the increasing expression of two common markers, NANOG and SOX2, during cervical squamous carcinogenesis and we confirmed their role in the progression of SIL to CSCC.

The most prevalent HPV type 16 in women with normal cervical cytology, precancerous cervical lesions and invasive cervical cancer was also the most common HPV genotype in our study.^44^ Infection with at least one HPV genotype was confirmed in all patients, and at least one HR-HPV genotype was present in 37 of 40 (92.5%) patients. As coinfection with more than one HPV genotype is a common event, we detected multiple HPV types in 45% of all patients, as expected.^45,46^ The majority of cervical SCC are HPV-related and develop from HSIL as a consequence of uncontrolled expression of the HR-HPV viral oncogenes E6 and E7.^5,7^ Recently, HR-HPV E6 and E7 oncoproteins have been reported to increase the expression of stem cell genes OCT3/4, NANOG and SOX2 and promote cell self-renewal upon inactivation of tumour suppressor proteins p53 and Rb.^47–51^ Other studies described a regulatory effect of NANOG and SOX2 on the expression of HR-HPV E6 and E7 oncogenes, so research regarding CSC markers and HPV infection is needed.^52,53^ Unfortunately, due to the small number of cases without HR-HPV infection, further analysis of NANOG and SOX2 expression between HR-HPV and non-HR-HPV group was not feasible in our present work.

NANOG is expressed in a variety of human malignancies, particularly breast cancer, colon cancer, head and neck cancer, lung cancer and pancreatic cancer. High levels of NANOG have been associated with metastatic disease and poor prognosis.^14,18^ While several studies have been conducted on NANOG in various organs, there are limited data on its expression in cervical squamous neoplasia.

In the present study, we observed cytoplasmic pattern of NANOG staining in the cervical squamous epithelium and associated neoplastic changes, whereas previous studies in the field reported cytoplasmic and/or nuclear expression.^33,34,36^ In our research group, predominantly cytoplasmic NANOG staining was observed in mild squamous dysplasia (very weak staining intensity) and in severe dysplasia (strong staining intensity) in head and neck squamous neoplasia.^37^ However, as a transcription factor, NANOG would be expected to be localized in the nucleus to function and accordingly, its nuclear pattern of expression has been observed in germ cell tumours.^37,54^ In other organs, NANOG has been detected mainly in the cytoplasm or mainly in the nuclei of malignant tumours, however some studies demonstrated NANOG in both sites of the cell.^18^ Protein modification and/or spatial structure changes could be one of the factors involved in cellular translocation and different distribution patterns of NANOG protein and different staining patterns consequetally.^34^ The cellular translocation of NANOG protein from nucleus to cytoplasm could be related to the molecular characteristics of the human NANOG protein, which has a region for nuclear localization and another region for nuclear export, suggesting a shuttling mechanism between nucleus and cytoplasm.^55^ Moreover, different staining patterns of NANOG could be furtherly explained by an antibody-dependent mechanism. Different antibodies were used in studies and some of them could possibly bind to the nuclear NANOG protein and some to the exported NANOG protein, or both.^34,56^

We found higher expression of NANOG in all SIL categories and CSCC compared to adjacent nondysplastic squamous epithelium. We also found higher NANOG expression in HSIL compared to LSIL, however we did not observe any significant difference in NANOG expression between HSIL and CSCC (p-value = 0.078). Noh *et al*. previously reported increased expression of NANOG in HSIL compared to LSIL and in CSCC compared to HSIL. Similar to our findings, they also described higher expression of NANOG protein in HSIL and CSCC compared to normal cervical epithelium.^35^ Ye *et al*. also, demonstrated higher expression of NANOG in LSIL compared to normal cervical squamous epithelium, in HSIL compared to LSIL, and in CSCC compared to HSIL.^36^ The small number of cases in our study may explain that we did not find a statistically significant difference in NANOG staining between HSIL and CSCC.

SOX2 is a key transcription factor that is expressed during embryonic development.^16,57^ Amplification of the SOX2 gene locus, leading to an increased expression, is supposed to be an important factor in cancerogenesis, especially in the pathogenesis of squamous cell carcinomas of various sites.^19,20^

In our study, SOX2 expression was localised in the nuclei of cervical squamous epithelium and associated neoplastic changes, as previously reported.^19–32^ In the non-dysplastic (non-atrophic and non-metaplastic) cervical squamous epithelium, we observed a consistent, moderate intensity of positive staining in basal cells, concordant with previous reports.^19,21,23,26,30^ Therefore, we considered SOX2 expression in the upper layers of squamous epithelium as abnormal and evaluated the intensity of SOX2 staining and the percentage of positive cells only above the basal layer.

We found increased expression of SOX2 in all grades of SIL and in CSCC, which is consistent with the findings by Kim *et al*.^28^ However, most other studies were mainly focused on the expression of SOX2 in CSCC and/or HSIL/CIN 3 and were reporting its increased expression in these neoplastic lesions.^19,21-24^ In our study, we observed an increase in SOX2 expression from LSIL to HSIL. In addition, we showed a significantly higher expression of SOX2 in HSIL/CIN 3 compared to HSIL/CIN 2. Kim *et al*. reported a gradual increase in SOX2 expression from normal cervical squamous epithelium to LSIL and HSIL, but no comparison was made between HSIL/CIN 2 and HSIL/CIN 3 categories.^28^ Additionally, they reported higher SOX2 expression in CSCC than in HSIL, which was also confirmed by Atigan *et al*.^28,31^ However, in our study, we found no difference in SOX2 expression between HSIL/CIN 3 or HSIL and CSCC, which is consistent with some other studies.^22,23,29^ Later research encompassing cervical squamous dysplasia and CSCC confirmed higher SOX2 expression in HSIL than in LSIL. A higher percentage of SOX2 nuclear staining in HSIL than in LSIL was described by Atigan *et al*.^31^ Wolsky *et al*. demonstrated statistically significant difference in SOX2 distribution between LSIL and HSIL, with similar results displayed by p16 and Ki-67. They even suggested that SOX2 is a diagnostic marker comparable to p16 and Ki-67 for distinguishing between LSIL and HSIL.^29^

The current WHO recommendation for cervical cytological or tissue specimens is to use a twotiered classification of squamous lesions, namely LSIL and HSIL terminology reflecting HPV-driven pathogenesis and establishing improved reproducibility of the LSIL/HSIL classification system.^5,6^ In HSIL category, lesions corresponding to HSIL/CIN 2, can be difficult to distinguish from LSIL. Block-type positive p16 staining supports the categorization of HSIL/CIN 2, while diagnosis of HSIL/CIN 2 is unlikely in the absence of p16 block-type positivity.^6^ On the other hand, up to 50% of LSILs could be block-type p16 positive.^11^ In the present study, we observed p16 positivity in the entire lesion in 2 of 10 LSILs and focal block-type p16 positivity next to heterogeneous or negative staining in 6 of 10 LSILs. In unequivocal LSILs, such p16 positivity is not conclusive for HSIL/CIN 2.^6,11,12^ Results of our study suggest NANOG and SOX2 as supportive diagnostic markers for the differentiation between LSIL and HSIL, which was proposed in some previously published studies.^28,29,31,35,36^

Higher expression of NANOG and SOX2 in LSIL compared to non-dysplastic cervical squamous epithelium could also help to distinguish between reactive changes and LSIL.^28,36^ Reactive or inflammatory features of non-dysplastic cervical squamous epithelium can mimic LSIL, resulting in overdiagnosis of LSIL in this context.^58,59^ The biomarker p16 has no utility at this diagnostic interface, as many LSILs are p16-negative. Moreover, the use of p16 is not recommended to diagnose LSILs with typical morphology.^6^ Therefore, past research and our data offer an opportunity to NANOG and SOX2 in that field. Areas of immature squamous metaplasia were previously described unstained with SOX2, and in our case of atrophic squamous epithelium there was no NANOG staining.^26,30^ Nevertheless, the significant difference in NANOG and SOX2 (SIxT score) expression between LSIL and non-dysplastic cervical squamous epithelium found in our study predict a potential of those two markers to identify LSIL. However, as it is a common diagnostic problem to distinguish between reactive changes and LSIL, further studies that include reactive or inflamed cervical squamous epithelium are needed.^6,58,59^

In general, all HSILs are treated by excision, as there are currently no reliable biomarkers for predicting the progression or regression of HSIL.^11–13^ However, it is known that HSIL/CIN 2 has a significantly higher regression rate than HSIL/CIN 3.^9^ Therefore, young women who wish to preserve fertility could be treated less aggressively.^5,40^ Accurate differentiation between HSIL/CIN 2 and HSIL/CIN 3 can be challenging and p16 immunostaining is not helpful as it is positive for both, HSIL/CIN 2 and HSIL/CIN 3 in most cases.^6,58^ In our study, we found a significantly higher SOX2 expression in HSIL/CIN 3 compared to HSIL/CIN 2, indicating a diagnostic potential of SOX2 as a biomarker in diagnostically challenging cases in this context. A specific expression pattern of SOX2 for HSIL/CIN 3 was also described by Moshi *et al*., providing a possibility for SOX2 to identify HSIL/CIN 3. Unfortunately, the distribution pattern of SOX2 staining throughout SIL in their study was sometimes complex, with a mixed and discontinuous pattern, consequentially limiting the use of SOX2 for recognizing HSIL/CIN 3.^32^ However, studies including more cases are needed to further assess the value of CSC markers in routine practice.

The present study has several strengths and limitations. The main strength is our systematic approach and the inclusion of the comparable number of cases in all SIL categories and CSCC. This enabled insight into NANOG and SOX2 expression during the whole process of cancerogenesis. Another strength is the fact that in addition to staining intensity and percentage of positive cells, we also evaluated the thickness of staining in squamous epithelium for NANOG and SOX2. We believe that immunohistochemical markers regarding cervical SIL should be assessed by the thickness of staining in the epithelium. Our belief is supported by at least two facts. First, the current WHO categorization of SIL is based on the thickness of dysplastic changes in epithelium.^5^ In addition, several previous studies have evaluated different markers according to the thickness of staining in squamous epithelium and specifically, p16 positivity in cervical SIL is defined as continuous staining in at least basal third of the epithelium.^6,29,40^ Another strength of our study is objective evaluation system for both NANOG and SOX2. We used the same criteria for the percentage of positive cells as some previously published studies, allowing a good comparison.^21,24,34,37^ We also established strict criteria for NANOG and SOX2 staining intensity, which were mostly undefined in previous studies. In contrast, the personal decision for assessment of NANOG and SOX2 staining intensity in a maximally stained area is the main limitation of our study. This could potentially overestimate the immunohistochemical expression of both markers. However, scoring in a maximally stained area is less subjective than scoring of the entire lesion. The small number of involved cases also limits the interpretations of our results. We only focused on immunohistochemical analysis of NANOG and SOX2 expression, which is another limitation. Further molecular studies of NANOG, SOX2 and their regulation need to be performed to understand their exact role in cervical carcinogenesis.

## Conclusions

We have shown an increased NANOG and SOX2 expression in LSIL, HSIL and CSCC compared to non-dysplastic cervical squamous epithelium. Our findings confirm the potential of NANOG and SOX2 in distinguishing LSIL from HSIL as surrogates for p16. NANOG and SOX2, in contrast to p16, could be suitable for the identification of LSIL in a background of cervical squamous epithelium exhibiting non-neoplastic changes mimicking SIL/CIN. In addition, SOX2 could be a marker for differentiation of HSIL/CIN 2 from HSIL/CIN 3. A clear advantage of SOX2 compared to NANOG and p16 is its uniform and easily interpretable nuclear staining. Further studies with larger numbers of patients and molecular insights should focus on the diagnostic and prognostic significance of NANOG and SOX2 in the entire process of cervical carcinogenesis.
